# Sphingomonas paucimobilis Bacteremia in a Patient With Retropharyngeal Abscess

**DOI:** 10.7759/cureus.25407

**Published:** 2022-05-27

**Authors:** Emmanuel Tito, Aamina Ahmad, Julita Gongolli, Wilo Issack, Alec Johnson

**Affiliations:** 1 Internal Medicine, West Virginia School of Osteopathic Medicine, Lewisburg, USA; 2 Internal Medicine, Western Michigan University Homer Stryker M.D. School of Medicine, Kalamazoo, USA

**Keywords:** oral infection, gram-negative bacteremia, hospital based, pharyngeal abscess, environmental bacteria

## Abstract

*Sphingomonas paucimobilis* is a nonfermenting gram-negative bacillus that is widely distributed in both community environments and hospitals. Various infections have been identified in humans, but most have been limited to case reports. When reported, it is most commonly nosocomial infections associated with contaminated hospital equipment such as indwelling catheters, ventilators, hemodialysis devices, and very rarely upper respiratory tract infections. We report an unusual presentation of *S. paucimobilis* infection. This case report describes a 59-year-old immunocompetent man who presented with a retropharyngeal abscess. Blood culture was positive for *S. paucimobilis*. The patient was treated for a total of 21 days of intravenous (IV) cefepime and oral (PO) metronidazole. He showed significant improvement and was discharged home with no medical sequelae.

## Introduction

*Sphingomonas paucimobilis* is a nonfermentative gram-negative rod of low pathogenicity. It affects primarily immunocompromised individuals and responds well to antibiotics [1]. The gram-negative, aerobic rod can be found naturally in soil and water sources, but the majority of infections are hospital-acquired due to contaminated water, drugs, or equipment [1,2]. Infection can occur in the setting of pneumonia, catheter-related bacteremia, peritonitis, meningitis, and soft tissue infection, although it is often difficult to isolate the source [1,2]. This paper highlights the first known case of *S. paucimobilis* septicemia in the setting of a retropharyngeal abscess.

## Case presentation

The patient is a male in his 50s with a history of a tobacco use disorder, chronic obstructive pulmonary disease, and hyperlipidemia who presented with severe throat and neck pain. The patient reported difficulty swallowing and pain with oral intake. The patient’s symptoms included globus sensation, nausea, shortness of breath, and one episode of emesis. He reported his symptoms began earlier in the day of admission, shortly after consuming lunch. He denied chest pain, changes in speech or ambulation, dizziness, fever, weight changes, numbness, and antecedent food-related trauma. The patient reported taking a home COVID test that was negative a day prior to admission. Of note, he had known poor dentition but had not sought dental care due to his recent loss of health insurance. He works as a carpenter, and he is routinely exposed to mold during construction. In the emergency department, the patient’s initial vital signs were within normal limits: blood pressure of 132/78 mmHg, heart rate of 97 beats per minute, respiratory rate of 16 breaths/minute, the temperature of 98.6 Fahrenheit (F), and oxygen saturation of 96% in ambient air. Examination findings included poor dentition with several dental cavities, posterior oropharyngeal erythema, mild abdominal tenderness, subcostal retractions, and rhonchorous breath sounds. Cardiovascular, neurological, and skin exams were unremarkable.

Over the course of several hours in the emergency department (ED), the patient developed stridor and vocal changes. His respiratory status progressively declined to the point of requiring endotracheal intubation for airway protection. He was immediately started on dexamethasone and broad-spectrum antibiotics with IV ceftriaxone and vancomycin after blood cultures were drawn. He was subsequently transferred to the medical intensive care unit (MICU) for further evaluation and treatment. 

At admission, laboratory studies showed increased leukocytosis, decreased hemoglobin, decreased sodium level, and slightly increased procalcitonin. Influenza A/B, respiratory syncytial virus (RSV), and COVID-19 polymerase chain reaction (PCR) were negative. The HIV test was negative. Liver enzymes, platelet count, total protein, albumin, total bilirubin, lactic acid, and thyroid-stimulating hormone levels were in the reference range. These results are indicated in Table 1. One out of two blood cultures returned positive for *S. paucimobilis* (time to detection was 25 hours). The second blood culture set did not yield any bacterial growth. Right upper quadrant ultrasound for the biliary source of infection was negative for any acute process.

**Table 1:** Laboratory studies. AST: aspartate aminotransferase, ALT: alanine aminotransferase, and TSH: thyroid-stimulating hormone.

Test	Patient’s values	Reference range	Units
White cell count	11.5 × 10^9^	4.0-11.0 × 10^9^	Cells//L
Hemoglobin	11.4	13.5-17.5	g/dL
Mean corpuscular volume	96.6	80-99	fL
Platelet count	350 × 10^9^	140-440 × 10^9^	Cells/L
Sodium	131	135-145	mmol/L
Procalcitonin	0.93	<0.50	ng/mL
AST	34	0-37	U/L
ALT	33	6-45	U/L
Total protein	6.9	6.4-8.3	g/dL
Albumin	4.0	3.5-5.0	g/dL
Total bilirubin	0.3	0.0-1.2	mg/dL
Lactic acid	0.7	0.7-2.5	mmol/L
TSH	2.60	0.27-4.20	uIU/ML

**Figure 1:**
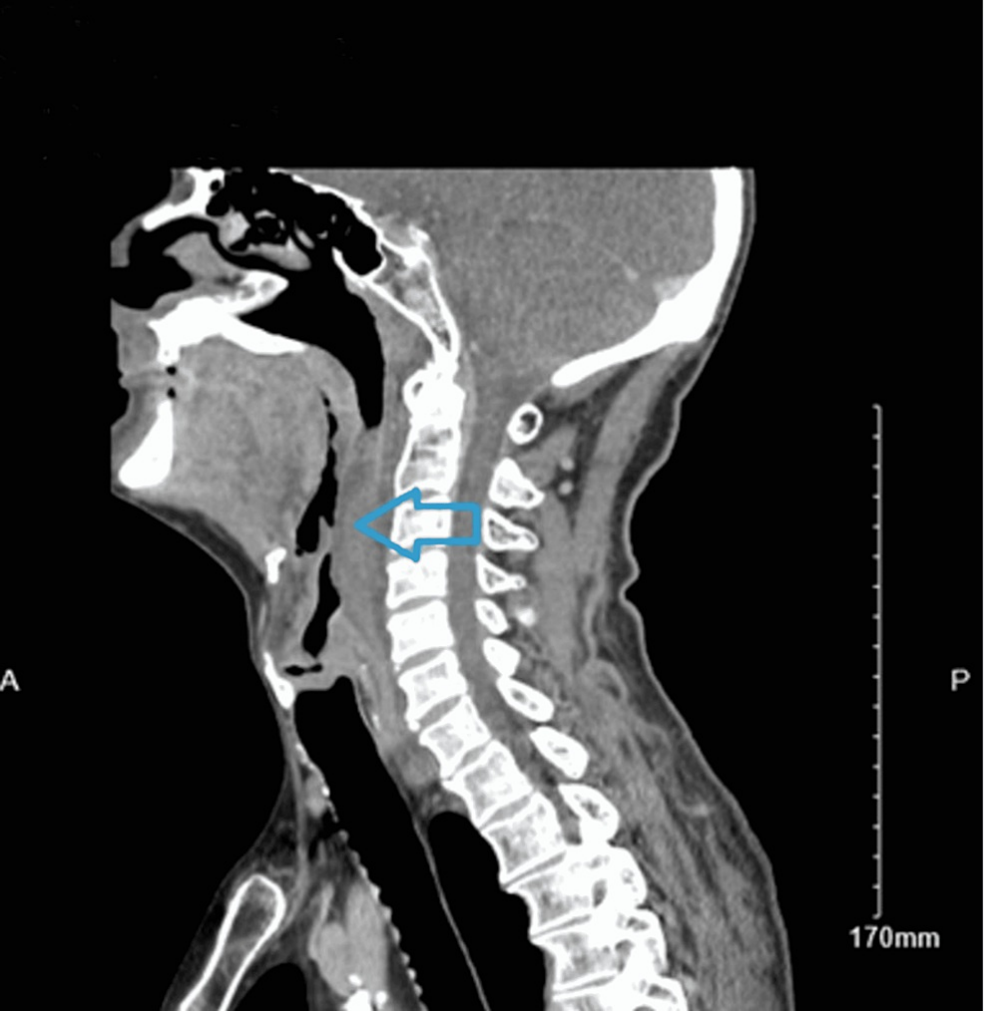
CT soft tissue neck with contrast revealing extensive asymmetric left retropharyngeal edema extending from the level of the oropharynx to the level of the larynx. There is a small 3 mm phlegmon and a marked narrowing of the hypopharyngeal airway (blue arrow). CT: computed tomography.

**Figure 2:**
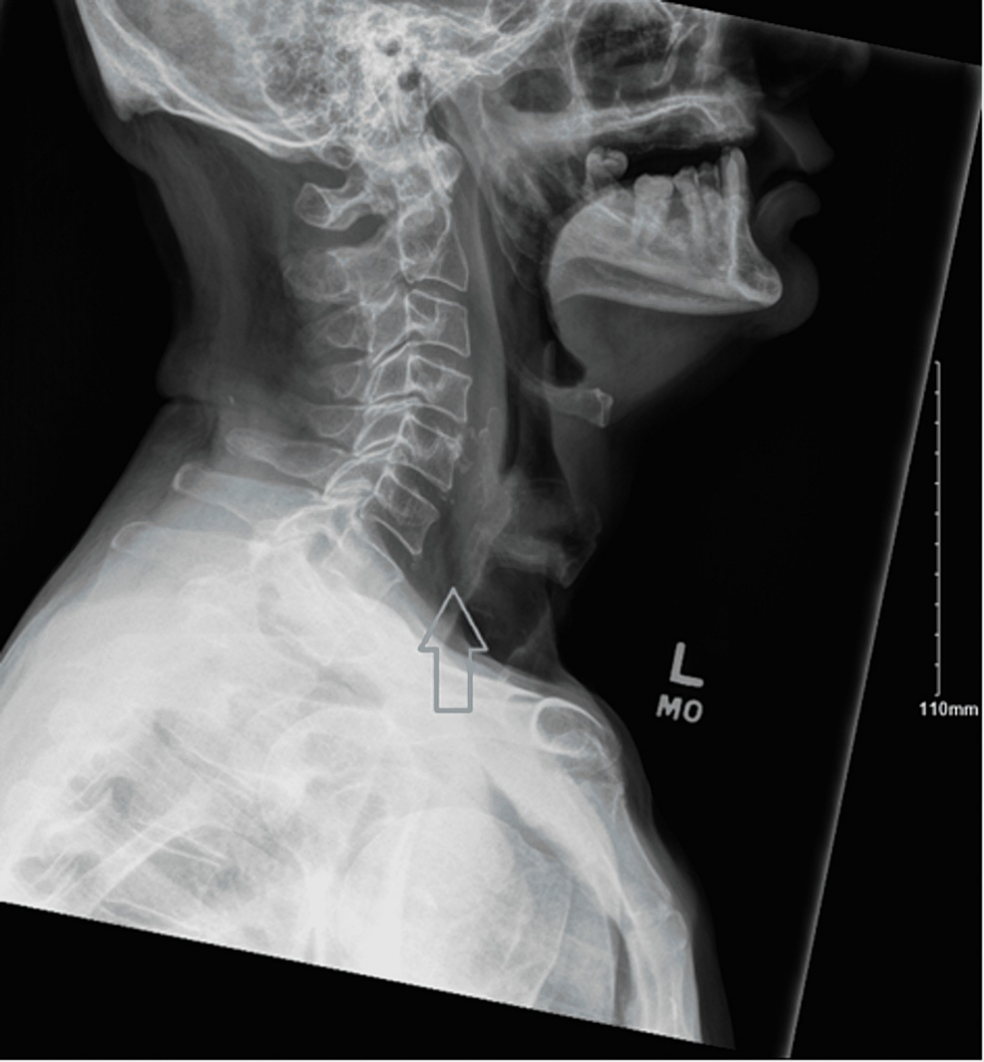
X-ray soft tissue neck revealing mild prevertebral soft tissue swelling at the level of C6 (grey arrow).

Based on the patient’s initial presentation, acute upper airway obstruction was the top differential. Other possible explanations for the patient’s symptoms at the time of presentation included community-acquired pneumonia, foreign body aspiration, esophageal tear or rupture, epiglottitis, and laryngeal malignancy-all being less likely given the clinical history, lack of systemic symptoms, lab results, and sudden onset. An upper airway mass was suspected due to his history of associated dysphagia, neck pain, shortness of breath, and acute onset. An x-ray of the soft tissue neck was done urgently, and the plain film demonstrated prevertebral soft tissue swelling at the level of C6 as seen in Figure 1. Subsequent computed tomography (CT) of the soft tissue neck with contrast confirmed extensive retropharyngeal edema spanning from the oropharynx to the larynx with a 3 mm phlegmon or abscess developing at the level of the oropharynx as seen in Figure 2.

The patient was initially started on empiric therapy for retropharyngeal abscess, including IV ceftriaxone, vancomycin, metronidazole, and dexamethasone. Surgical drainage was avoided due to the lack of adequate access to the retropharyngeal space and rapid clinical improvement. Two days later, he was extubated and transferred out of the MICU. His antibiotic regimen was de-escalated to IV cefepime and metronidazole after confirmation of *S. paucimobilis*. The patient gradually improved with repeat soft tissue neck CT, demonstrating a significant decrease in airway swelling and abscess resolution. He continued to improve on the antibiotic regimen. Repeat blood cultures did not yield any bacterial growth. The patient was hospitalized for eight days and discharged with a peripherally inserted central catheter (PICC) to complete 21 days of antibiotics. He was discharged with no apparent medical sequelae.

## Discussion

*S. paucimobilis* is an aerobic, gram-negative rod belonging to the *Sphingomonas* genus [1,3]. Previously known as *Pseudomonas paucimobilis*. It is commonly found in soil and water, including water systems where it can form significantly dense biofilms on pipes and filters, and hospital materials such as contaminated drug solutions and dialysis fluid [1-4]. It is thought to be an organism of low virulence but can cause infection in humans, primarily in immunocompromised individuals as nosocomial infections [1]. Cases of *Sphingomonas* infection include catheter-related bacteremia, contaminated intravenous antibiotics, pneumonia, meningitis, soft tissue infections, urinary tract infections (UTIs), and wound infections among others [1,2].

While gram-negative bacteria are common culprits of hospital-acquired infections, *Sphingomonas* is unique because it does not elicit a strong immune response from the host [4]. It has a specific sphingolipid structure that is less activating to host monocytes, resulting in a weaker immune response [4,5]. This is believed to be why cases of *Sphingomonas* infection often present with vague symptoms and how bacteria often escape into the bloodstream [4,5].

In a study examining documented infections due to *S. paucimobilis* between 1979 and 2009, a total of 52 cases were identified, of which the most common presentations included bacteremia or sepsis, peritonitis, lung infection, and UTI [2]. Many of these patients (46%) developed hospital-acquired *Sphingomonas* infection, and 50% of cases were due to an unidentified source [2]. Patients with *Sphingomonas* infection improve with antibiotics and source control. Possible antibiotic options include broad-spectrum beta-lactam antibiotics, beta-lactams with beta-lactamase inhibitors, cephalosporins, fluoroquinolones, and carbapenems [1]. However, it is not uncommon for clinicians to choose more aggressive treatment consisting of numerous agents as affected patients often have significant comorbidities [4]. Some studies show resistance to penicillin and first-generation cephalosporin because of the production of chromosomally encoded β-lactamase production [6,7]. Thus, there are no definitive guidelines for an antimicrobial regimen for *S. paucimobilis*; treatment is done with individualized antibiotic therapies according to the in vitro susceptibility profile of the clinical isolate and patient characteristics [8].

At the time of presentation, our patient’s retropharyngeal abscess was unable to be drained and cultured because it had significantly reduced in size on repeat imaging. The definitive source of this patient’s *Sphingomonas* infection remains unclear, but it was thought to be from his abscess. A nosocomial source from his endotracheal tube could not be excluded.

## Conclusions

Two weeks after discharge, he followed up with an otolaryngologist and reported gradual resolution of symptoms. Laryngoscopy showed detailed visualization of the patient’s glottic, supraglottic, and pharyngeal anatomy without obstruction. He did not have any sequelae noted during follow-up. This report documents a case of *S. paucimobilis* septicemia in a patient with a retropharyngeal abscess. *S. paucimobilis* can lead to infections in both immunocompromised and healthy individuals. Despite being an organism of low clinical virulence, the infection caused by *S. paucimobilis* can lead to severe sepsis.
